# Vegetarian Nutrition in Chronic Kidney Disease

**DOI:** 10.3390/nu16010066

**Published:** 2023-12-25

**Authors:** Yoko Narasaki, Kamyar Kalantar-Zadeh, Connie M. Rhee, Giuliano Brunori, Diana Zarantonello

**Affiliations:** 1 Department of Medicine, David Geffen School of Medicine at UCLA, Los Angeles, CA 90073, USA; 2 Tibor Rubin Veterans Affairs Long Beach Healthcare System, Long Beach, CA 90822, USA; 3 The Lundquist Institute at Harbor-UCLA Medical Center, Torrance, CA 90502, USA; 4 Division of Nephrology, Hypertension, and Kidney Transplantation, University of California Irvine, Orange, CA 92868, USA; 5 Nephrology Section, Veterans Affairs Greater Los Angeles Healthcare System, Los Angeles, CA 90073, USA; 6 Department of Nephrology, Santa Chiara Hospital, APSS, 31822 Trento, Italy; 7 CISMed, University of Trento, 38122 Trento, Italy

**Keywords:** nutrition, vegetarian diet, plant-based diet, chronic kidney disease

## Abstract

There is rising interest globally with respect to the health implications of vegetarian or plant-based diets. A growing body of evidence has demonstrated that higher consumption of plant-based foods and the nutrients found in vegetarian and plant-based diets are associated with numerous health benefits, including improved blood pressure, glycemic control, lipid levels, body mass index, and acid–base parameters. Furthermore, there has been increasing recognition that vegetarian and plant-based diets may have potential salutary benefits in preventing the development and progression of chronic kidney disease (CKD). While increasing evidence shows that vegetarian and plant-based diets have nephroprotective effects, there remains some degree of uncertainty about their nutritional adequacy and safety in CKD (with respect to protein-energy wasting, hyperkalemia, etc.). In this review, we focus on the potential roles of and existing data on the efficacy/effectiveness and safety of various vegetarian and plant-based diets in CKD, as well as their practical application in CKD management.

## 1. Introduction

There is rising interest worldwide regarding the health implications of vegetarian or plant-based diets, including reductions in animal-based food intake and/or fully excluding animal-based products from the diet [1]. A growing body of evidence has demonstrated that higher consumption of plant-based foods and the nutrients found within plant-based diets are associated with numerous health benefits, including improved blood pressure, glycemic control, lipid levels, body mass index (BMI), and acid–base parameters, as well as lower risk of complications such as diabetes [2], cardiovascular disease [3], and death [4]. Furthermore, there has been increasing recognition that plant-based diets have a potential salutary role in the management of chronic kidney disease (CKD). For example, the low-protein vegan diet (0.7 g/kg of body weight/day of protein), the low-protein supplemented vegan diet (0.6 g/kg of body weight/day of protein supplemented with essential amino acids (EAAs) and keto acids (KAs), i.e., one tablet per 10 kg of body weight), and the very-low-protein diet (0.3 g/kg of body weight/day of protein supplemented with EAAs and KAs, i.e., one tablet for every 5 kg of body weight) are vegan/vegetarian diets that have been proposed as possible kidney-conservative treatments [5]. A tablet of Ketosteril^®^, which is used globally, contains L-lysine (105 mg), L-threonine (53 mg), L-histidine (38 mg), L-tyrosine (30 mg), L-tryptophan (23 mg), hydroxy-methionine (59 mg), calcium-keto-valine (86 mg), calcium-keto-phenylalanine (68 mg), calcium-keto-leucine (101 mg), and calcium-keto-isoleucine (67 mg) [6]. Moreover the “Plant-Dominant Low-Protein Diet” (PLADO) [7] and “Plant-Focused Nutrition in Patients With Diabetes and CKD Diet” (PLAFOND) [8] are two subtypes of plant-based diets that have been established for people with CKD as a means to reduce the progression of kidney disease (Table 1). A sizeable body of research has shown that vegetarian diets have nephroprotective effects, although there remains some degree of uncertainty about safety with respect to the high contents of minerals such as phosphorus and potassium, along with the potential risks of hyperphosphatemia and/or hyperkalemia that may ensue with greater plant-based food consumption. In this review, we focus on the potential roles of and existing data on vegan, lacto-ovo vegetarian, and PLADO diets in CKD, as well as the practical application of these diets in CKD management.

## 2. Overview of Vegetarian and Plant-Based Diets

**Figure 1:**
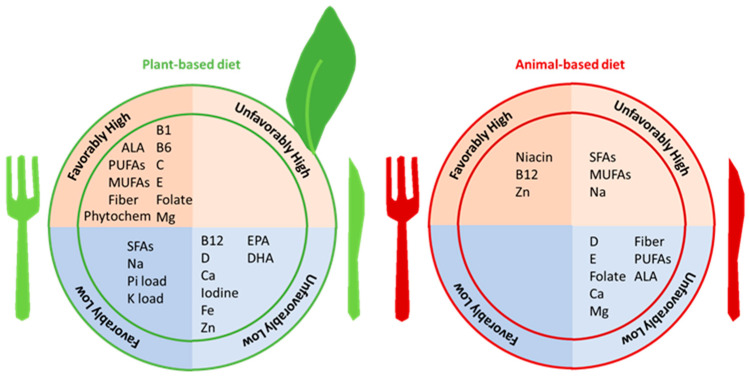
Characteristics of nutrients and components in plant-based vs. animal-based diets. Abbreviations: ALA, α-linolenic acid; B1, vitamin B1; B6, vitamin B6; B12, vitamin B12, Ca, calcium; D, vitamin D; DHA, docosahexaenoic acid; EPA, eicosapentaenoic acid; Fe, iron; K, potassium; Mg, magnesium; MUFAs, monounsaturated fatty acids; phytochem, phytochemicals; Pi, phosphorus; PUFAs, total polyunsaturated fatty acids; SFAs, saturated fatty acids; Zn, zinc.

Vegetarian or plant-based diets are types of diets composed of a larger proportion of foods from plant-based sources as opposed to animal-based sources. There are various forms of vegetarian diets, such that some types fully exclude all animal products (i.e., vegan diets), whereas other types include dairy products such as milk and cheese, eggs, and honey (i.e., lacto-ovo vegetarian diets) or may even include small amounts of fish and seafood (i.e., pescatarian), as well as meat and poultry (i.e., semi-vegetarian or flexitarian) [11]. The phrases “vegetarian” and “plant-based diet” are often used without differentiation, but the terminology “vegetarian” is commonly used to refer to lacto-ovo vegetarians, while the terminology “plant-based diet” is used to refer to dietary patterns with a greater proportion of foods derived from plant-based sources but may not mean that they are devoid of animal-based foods. In other words, a plant-based diet is a hybrid form of a diet rich in plant-based foods. A person who consumes a plant-based diet eats healthy plant-based foods (i.e., fresh/whole/unprocessed/unrefined foods and beverages) and avoids unhealthy plant-based foods (i.e., processed/refined/sugar-sweetened foods and beverages) [12]. Two types of plant-based diets that have specifically been designed for the non-dialysis-dependent CKD (NDD-CKD) population include the (1) Plant-Dominant Low-Protein Diet (PLADO), consisting of a dietary protein intake of 0.6–0.8 g/kg/day, with >50% from plant-based sources [7], and the (2) Plant-Focused Nutrition in CKD and Diabetes Diet (PLAFOND), consisting of a dietary protein intake of 0.6–0.8 g/kg/day from >50% plant-based sources [8]. Low-protein diets are supported by clinical practice guidelines to ameliorate the progression of CKD, and they are considered to be the centerpiece of conservative and preservative kidney disease management strategies as a means to delay or avert the need for dialysis [7,8]. Irrespective of the specific type of plant-based diet, such diets typically consist of a greater proportion of healthy plant-based foods (i.e., whole grains, cereals, nuts, fruits, and vegetables) and favorable nutrient profiles (i.e., dietary fiber, unsaturated fatty acids, folate, magnesium, vitamin C, vitamin E, carotenoids, phytochemicals, and low bioavailability of phosphorus and potassium) (Figure 1) [11]. Dietary phosphorus and potassium from unprocessed plant-based foods have lower bioavailability and, therefore, confer lower loads of phosphorus and potassium, respectively, compared to animal-based foods and processed foods, which is in part due to concomitantly higher glucose and dietary fiber contents. Additionally, phosphorus in plant-based foods is present in the form of phytate, which generally has limited bioavailability in the human digestive system. There is more discussion on this topic in the latter part of this review.

**Figure 2:**
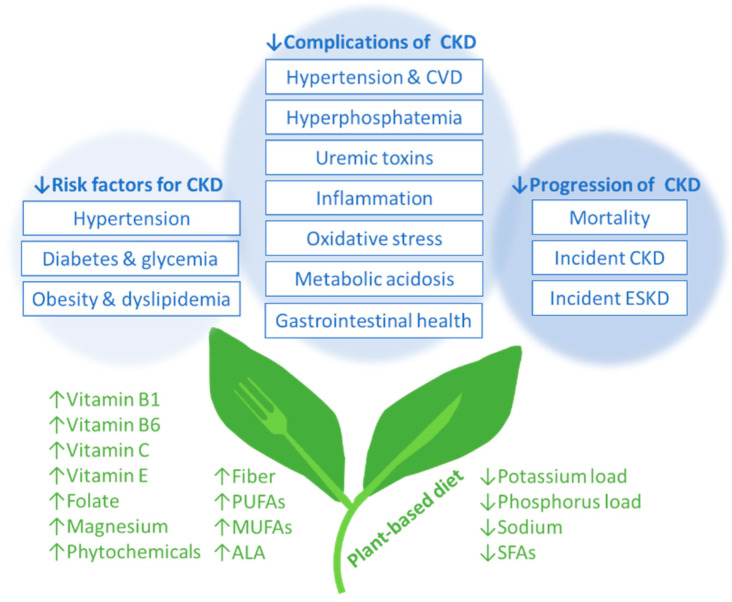
Plant-based diets and health benefits in chronic kidney disease. Abbreviations: ALA, α-linolenic acid; CKD, chronic kidney disease; CVD, cardiovascular disease; ESKD, end-stage kidney disease; MUFAs, monounsaturated fatty acids; PUFAs, total polyunsaturated fatty acids; SFAs, saturated fatty acids.

In the general population, the popularity of plant-based diets is in part due to their perceived health benefits related to the control of diabetes, obesity, hypertension, and hyperlipidemia. More recently, there has been interest in the role of plant-based diets in preventing the development of de novo CKD, attenuating CKD progression, and mitigating CKD-related complications (Figure 2).

## 3. Vegetarian Diets and Risk Factors for Incident CKD

### 3.1. Hypertension in Non-CKD Populations

Hypertension and CKD are closely interrelated, such that sustained hypertension can lead to incident CKD and CKD progression, which can in turn result in worse blood pressure (BP) control [13]. Randomized controlled trials have shown the benefits of plant-based diets for BP control. In a study of 59 normotensive participants without underlying CKD, consumption of a vegetarian diet for a six-week period lowered their mean systolic BP by 6.8 mmHg when measured at the laboratory, and by 4.9 mmHg when measured at home [14]. Another study in 58 participants with mild untreated hypertension, comparing ovo-lacto-vegetarian vs. omnivorous diets, showed that the ovo-lacto-vegetarian diet resulted in a reduction in BP by an average of 5.5 mmHg [15]. The Dietary Approach to Stop Hypertension (DASH) trial, a landmark randomized controlled trial examining the effects of a largely plant-based diet on BP control, showed that the DASH diet reduced BP by an average of 5.5 mmHg compared to the control diet [16]. A meta-analysis of seven clinical trials with an aggregate of 313 participants, which excluded the DASH diet trials, also confirmed the benefits of plant-based diets for BP, such that consumption of vegetarian diets reduced systolic BP by a mean of 4.8 mmHg compared to omnivorous diets [17].

### 3.2. Diabetes Mellitus in Non-CKD Populations

Vegetarian diets have been reported as an effective intervention for the prevention and treatment of diabetes mellitus, the dominant etiology of CKD globally. It has been shown that the prevalence of type 2 diabetes in people consuming vegetarian diets is lower than that among non-vegetarians, even after adjusting for BMI [18]. A meta-analysis of nine large prospective studies with a total of 307,099 participants reported an inverse association between higher adherence to a plant-based dietary pattern and the risk of type 2 diabetes [19]. This association was strengthened in healthy plant-based diet patterns, i.e., consumption of more healthy plant-based foods (e.g., whole grains, fruits, vegetables, nuts, legumes, vegetable oils, tea, and coffee) vs. unhealthy foods (e.g., fruit juices, refined grains, fried potatoes or potato chips, desserts, and sweetened beverages).

Several potential mechanisms explain the relationship between plant-based diets and lower risk of diabetes mellitus. For example, the foods in healthy plant-based diets individually and jointly reduce the risk of diabetes by improving insulin sensitivity and BP [17,20], mitigating long-term weight gain, and ameliorating systemic inflammation [21]. Moreover, plant-based diets may reduce the risk of type 2 diabetes by ameliorating excessive weight gain. Multiple interventional and observational studies have shown that plant-based diets provide favorable weight control and/or weight loss in the short term and weight loss and/or prevention of weight gain in the long term [22,23,24]. Plant-based diets may also improve circulating levels of adiposity-related biomarkers, including leptin, adiponectin, high-sensitivity C-reactive protein, and interleukin-6 [25,26].

## 4. Vegetarian Diets and CKD Complications

### 4.1. Hypertension in CKD Populations

Different components of vegetarian diets contribute to directly or indirectly lowering BP levels in people with CKD, through various pathways. First, lower consumption of sodium in plant-based vs. animal-based diets can prevent and control hypertension. Unprocessed plant-based foods generally have less sodium than animal-based foods and processed foods. Indeed, data from the National Health and Nutrition Examination Survey (NHANES) showed that vegetarians ate less sodium, as ascertained using 24 h dietary recall, compared to non-vegetarians (2347 ± 80 mg vs. 3621 ± 27 mg) [27]. A meta-analysis that included 21 studies among people with earlier stages of CKD, dialysis patients, and kidney transplant recipients reported that salt reduction reduced systolic and diastolic BP in the short term (i.e., 1 to 36 weeks) [28]. This study also reported that salt reduction resulted in lower albuminuria levels. Another meta-analysis also showed that salt restriction was associated with lower systolic BP, diastolic BP, and proteinuria levels among 738 people with stages 1–4 CKD [29], and another pooled analysis showed that reduction of salt intake resulted in lower systolic and diastolic BP among 101,077 people with CKD [30].

Second, higher potassium intake from plant-based diets may help reduce BP. It is well established that higher dietary potassium intake lowers BP in the general population. While studies examining the effects of dietary potassium on BP in people with CKD are sparse, limited data suggest potential benefits. In an animal study of rats with CKD, it was demonstrated that potassium supplementation lowered BP among rats with slightly higher serum potassium levels compared to rats on a low-potassium diet [31]. In a non-randomized study of 11 people with stage 3 CKD, receipt of the DASH diet (dietary potassium intake of 4.7 g/day) over two weeks resulted in no differences in clinical and mean 24 h ambulatory BP, whereas it resulted in lower nighttime systolic BP levels compared to BP levels during the baseline period while on a control diet (dietary potassium intake of 2.4 g/day) in the absence of hyperkalemia [32]. Randomized trials in people with stages 3–4 CKD have also shown that receipt of diets that are higher in fruits and vegetables resulted in lower systolic BP after one year [33] or three years [34], although narrowly missing statistical significance after a shorter follow-up period of four weeks [35].

Third, all plant-based foods contain dietary fiber, a carbohydrate that is indigestible by digestive tract enzymes (Table 2) [35]. Dietary fiber intake improves BP by modifying arterial contraction due to its effect on arterial smooth muscle, influencing the activity of the angiotensin-converting enzyme (ACE) or retaining minerals such as potassium and magnesium in its matrix [36,37]. In addition to BP control, there are a variety of health benefits of dietary fiber that affect CKD outcomes. For example, dietary fiber intake can improve glycemic control by delaying gastric emptying, reducing postprandial glucose absorption, providing a lower glycemic response, producing greater satiety, and improving insulin sensitivity [8,38]. Moreover, dietary fiber intake also contributes to improving dyslipidemia. Dietary soluble fiber with high viscosity decreases cholesterol absorption, binds to bile acids, and increases their fecal excretion. Bacterial fermentation in the colon can inhibit cholesterol production in the liver by producing short-chain fatty acids (SCFAs) [39]. SCFAs also exert trophic action on the mucosa and strengthen the defense function of the intestinal barrier by counteracting bacterial translocation and low-grade chronic inflammation [40]. Moreover, fiber intake reduces serum urea levels by promoting a fecal route of excretion for nitrogenous waste, and it can reduce serum levels of AGEs (advanced glycation end products) [35]. Lastly, greater dietary fiber intake may lead to improvements in constipation, increased satiety, reduced energy intake, weight control, and slower absorption of some nutrients in the intestine, leading to reduced inflammation [36].

Fourth, more balanced intake of macronutrients (including dietary protein, fat, and carbohydrates) conferred by a plant-based diet can contribute to better BP control. Results from observational studies indicate an inverse association between dietary plant protein intake and BP [41], and both prospective studies and randomized controlled trials have shown similar relationships between plant and animal protein intake with respect to BP [42]. The effects of plant vs. animal protein on BP control remain to be established. Additionally, vegetarian diets usually provide low intake of saturated fatty acids and omega-3 polyunsaturated fatty acids (PUFAs). In a cross-sectional study of 26 vegetarians vs. 26 non-vegetarians, matched according to age, sex, and BMI, the vegetarians had higher plant-based fat consumption than the non-vegetarians, which may lead to higher resting energy expenditure (REE) in vegetarians and potentially contribute to better body weight and BP control [43].

### 4.2. Hyperphosphatemia in CKD Populations

In people with advanced CKD, decreased phosphorus excretion by the kidneys, coupled with disordered mineral metabolism, engenders hyperphosphatemia, leading to vascular calcification and stiffness, altered cardiac structure and function, kidney osteodystrophy, and increased mortality [44]. Therefore, in the traditional dietary management of advanced CKD patients, dietary phosphorous has been restricted and plant-based foods have been avoided due to concerns regarding high contents of minerals such as phosphorus. However, increasing evidence suggests that greater intake of plant-based foods may lead to better phosphorus control. The amount of phosphorus contained in food vs. phosphorus absorbed by the body is not always consistent. Given that phosphorus in plant-based foods is often in the form of phytate (which humans have limited ability to digest, given the absence of the phytase enzyme), phosphorus found in plant-based foods usually has lower absorbability and/or bioavailability (20 to 40% bioavailability) compared with animal foods, which often have phosphorus in the form of caseins (40 to 60% bioavailability), and processed foods, in which phosphorus is usually present as food additives (~100% bioavailability) [45,46]. Indeed, both animal and human studies show reduced phosphorus loads when consuming plant-based vs. animal-based diets, despite both diets having the same amounts of phosphorus. In a rat model of CKD–mineral bone disease (CKD–MBD), administration of a plant-based diet led to a reduced phosphorus load, such that rats fed grain-based diets showed similar serum phosphorus levels, calcium levels, and intact parathyroid hormone (PTH) levels, yet lower urinary phosphorus excretion and serum fibroblast growth factor 23 (FGF-23) levels vs. rats fed the same amount of phosphorous from casein-based diets [47]. In a crossover trial of people with stage 3–4 CKD, receipt of a vegetarian diet for one week led to lower serum phosphorus, phosphaturia, and FGF-23 levels compared to a meat-based diet with the same phosphorus content [48]. A randomized controlled trial in which participants underwent partial replacement of animal protein with plant protein also led to reduced serum phosphorus levels [49].

### 4.3. Uremic Toxins, Inflammation, and Oxidative Stress in CKD

Given the concomitant rich consumption of dietary fiber, along with their lower contents of carnitine, choline, phosphatidylcholine, tyrosine, and tryptophan, plant-based diets lead to less generation of uremic toxins (i.e., trimethylamine n-oxide (TMAO), indoxyl sulfate, and p-cresyl sulfate), as well as reducing inflammation and oxidative stress [50,51]. In a randomized controlled study of 32 non-dialysis-dependent CKD patients, one week of a supplemented very-low-protein diet of plant-based origin (0.3 g/kg body weight/day) led to reduced indoxyl sulfate levels [52]. In a randomized controlled study of 40 hemodialysis patients who received higher vs. lower dietary fiber intake for six weeks, those who received higher dietary fiber intake had reduced free plasma levels of indoxyl sulfate and p-cresyl sulfate [53]. Data from the NHANES III cohort included 14,543 participants, in whom it was observed that dietary fiber intake was negatively associated with serum C-reactive protein (CRP) levels, such that each 10 g/day increase in total fiber intake was associated with an 11% and 38% decline in the odds of elevated serum CRP levels in the CKD and non-CKD groups, respectively [54]. In a rat model of CKD, consumption of high-amylose maize resistant starch for three weeks also ameliorated inflammation and oxidative stress [55].

Dietary fiber confers a number of advantages for sustainable human health [56]. The bulking effect from the food is important to control the events in the digestive tract, including improved gastrointestinal motility (i.e., increased bowel movements and reduced intestinal transit time), increased fecal bulk, and greater stool frequency. Dietary fiber adds bulk not only to stool, but also to the overall diet, which provides a satiety effect and regulates energy intake. This bulking property of dietary fiber can also reduce BP, promote weight loss, and alleviate constipation [57]. The viscosity effect of dietary fiber can also improve glycemic and cholesterol control, and it may additionally contribute to cancer prevention. Increasing viscosity during digestion due to soluble dietary fiber results in the trapping of carbohydrates, slowing of glucose absorption, and lowering of postprandial blood glucose levels. Soluble fiber also helps to reduce total and LDL cholesterol levels by binding bile acids in the small intestine following extraction from the body through feces, as well as increasing the synthesis of bile acids from cholesterol. Dietary fiber also traps carcinogenic substances and may prevent the development of cancer [57]. Fermentable dietary fiber is the substrate for bacterial metabolism and stimulates the production of short-chain fatty acids (SCFAs) through intestinal fermentation, primarily acetate, propionate, and butyrate, leading to its protective effects against inflammation, obesity, diabetes, cancer, and cardiovascular disease, along with immune regulation and a number of other health benefits [58,59]. The mechanisms underlying the association between dietary fiber intake and lower uremic toxin levels, as well as urea and creatinine concentrations, are interrelated, such that greater dietary fiber intake (1) decreases toxin absorption and increases their fecal excretion by improving intestinal motility, (2) reduces the permeability of toxins by improving the integrity of tight junctions in the colonic epithelium by producing SCFAs, and (3) facilitates the growth of a more favorable microbiome.

### 4.4. Metabolic Acidosis

A large proportion of people with CKD suffer from metabolic acidosis and its adverse consequences, including muscle wasting, bone loss, impaired insulin sensitivity, chronic inflammation, and progression of kidney disease [60]. While alkali therapy is typically conducted to correct metabolic acidosis in CKD patients by administering sodium bicarbonate, a series of trials have shown that plant-based diets could also be used to treat metabolic acidosis. In a randomized controlled trial of 71 people with stage 4 CKD, people assigned to greater fruit and vegetable intake over the course of one year had higher plasma CO_2_ levels and lower urinary indices of kidney injury [33]. Another randomized controlled trial of 108 people with stage 3 CKD also confirmed similar effects of fruit and vegetable intake on metabolic acidosis parameters, such that daily administration of two to four cups of fruits and vegetables over a period of three years resulted in higher CO_2_ levels, lower net acid excretion, lower urinary albumin–creatinine ratios, and preserved kidney function [34]. According to this evidence, the KDOQI guidelines also support prescribing more fruits and vegetables for stage 1–4 CKD patients in order to decrease their body weight, blood pressure, and net acid production [36].

## 5. Vegetarian Diets, Incident CKD, and CKD Progression

### 5.1. Incident CKD and CKD Progression

Several studies have shown favorable associations of plant-based diets with CKD outcomes, including incident CKD (i.e., development of albuminuria and/or eGFR decline) and CKD progression. With respect to the outcome of incident CKD, among participants in the Tehran Lipid and Glucose Study (TLGS), those in the highest quartile of plant protein intake exhibited a 30% lower risk of developing CKD than those in the lowest group of plant protein intake, while those in the highest quartile of animal protein intake had a 37% higher risk of de novo CKD than those in the lowest group of animal protein intake [61]. In the Multi-Ethnic Study of Atherosclerosis (MESA), a dietary pattern with higher intake of whole grains, fruits, vegetables, and low-fat dairy foods was associated with a 20% lower risk of CKD, whereas nondairy animal food intake was associated with an 11% higher urinary albumin-to-creatinine ratio [62]. In a large longitudinal observational study of the Atherosclerosis Risk in Communities (ARIC) cohort, which included 11,952 adults with normal kidney function at baseline, various sources of dietary protein intake had differential associations with the risk of CKD [63]. During a median follow-up of 23 years, there was a higher risk of incident CKD in those consuming greater amounts of protein from red and processed meat sources. Compared to those in the lowest quintiles of red and processed meat consumption, those in the highest quintile of intake had a 23% higher risk of incident CKD. Moreover, this study showed favorable associations of vegetable sources of proteins, such that those in the highest quintile of vegetable protein intake had a 24% reduced risk of incident CKD compared to those in the lowest quintile of intake. Furthermore, when one serving per day of nuts or legumes was used to substitute one serving per day of red and processed meat, a reduced risk of incident CKD was observed.

With respect to the outcome of CKD progression, in a prospective cohort study of approximately 1600 women from the Nurses’ Health Study (NHS), among those with mild CKD, greater intake of both total protein and nondairy animal protein was associated with a decline in eGFR over a follow-up period of 11 years (i.e., each increment of +10 g/day of total protein intake and nondairy animal protein intake was associated with an eGFR decline of −7.72 and −1.21 mL/min per 1.73 m^2^, respectively) [64]. Existing clinical trial data have also shown that partial replacement of animal protein with plant protein leads to reductions in albuminuria [49,65,66]. Finally, a recent systematic review suggested that a vegetarian diet improves renal filtration function in CKD patients [67].

### 5.2. Progression of ESKD

End-stage kidney disease (ESKD) necessitating long-term dialysis or kidney transplantation is another highly relevant outcome with respect to studying the impact of vegetarian diets on kidney health. There are mixed data, such that some studies have provided evidence that vegetarian diets are associated with a lower risk of incident ESKD [68,69], whereas others have not observed a nephroprotective relationship [70,71]. A report from the Singapore Chinese Health Study showed the deleterious impact of high red meat intake on progression to ESKD, and it also showed that substituting one serving of red meat with one serving of soy/legumes was associated with a lower risk of incident ESKD [69]. In contrast, a meta-analysis showed no statistically significant association between healthy dietary patterns (i.e., those higher in fruits and vegetables, fish, legumes, cereals, whole grains, and fiber; and lower in red meat, salt, and refined sugars) and risk of ESKD, due to the competing risk of death and the relatively small number of events [70]. Similarly, among 3972 people with CKD from the Reasons of Geographic and Racial Differences in Stroke (REGARDS) study, there were no significant associations between dietary patterns and the risk of incident ESKD in multivariable models adjusted for age, race, sex, geographic region of residence, and caloric intake, nor in models further adjusted for socioeconomic and lifestyle factors, comorbidities, and baseline kidney function [71]. One possible explanation for the lack of a nephroprotective association between plant-based diets and ESKD in these studies may relate to inadequate power due to the relatively modest number of ESKD events.

## 6. Practical Application of Vegetarian Diets in CKD

### 6.1. Protein-Energy Wasting

People with CKD are more predisposed to malnutrition–wasting conditions, including protein-energy wasting (PEW), which adversely impacts their health and survival [72]. The prevalence of PEW is increasingly higher with incrementally lower levels of kidney function, and more than half of people treated with maintenance dialysis therapy may suffer from this complication [73]. Thus, there has been concern about the potential nutritional adequacy of vegetarian diets in people with CKD, particularly with respect to energy and protein contents. However, a number of studies in experimental animal models [47,74,75] and human studies [76] have shown that vegetarian diets are indeed nutritionally adequate in CKD. For example, in a study of 239 people with advanced CKD, it was shown that vegetarian diets with very low protein contents (dietary protein intake of 0.3 g/kg/day) supplemented with keto analogues provided satisfactory nutritional status (i.e., BMI and serum albumin levels remained stable over a mean duration of 29.6 months) [70]. Another study of people with diabetes with elevated proteinuria levels demonstrated that consumption of a predominantly vegetable-protein diet (dietary protein intake of 0.7 g/kg/day) over eight weeks resulted in no considerable differences in body weight or triceps skinfold thickness [77]. Moreover, among people with diabetes, transitioning from a diet with a dietary protein intake of 1.0 to 1.3 g/kg/day to a vegan diet with a dietary protein intake of 0.7 g/kg/day was not associated with substantial changes in serum total protein or serum albumin levels [78]. Moreover a randomized controlled trial recently compared 43 people receiving a low-protein diet with soy protein (60% soy protein and 40% other vegetable proteins) plus KAs vs. 42 people who received a conventional low-protein diet and found that receipt of a low-protein diet with vegetable proteins and KAs was associated with a slower loss of lean mass [79]. Hence, growing research shows that people with CKD who consume vegetarian diets, including those on maintenance dialysis, are not at higher risk of PEW, although further investigation in this area is needed [80].

### 6.2. Overall Nutritional Adequacy

While plant-based diets are generally considered to be healthier, there are concerns as to whether these diets have adequate contents of nutrients that are typically found in animal-based foods (Table 3). However, ensuring nutritional adequacy is an issue not only in CKD populations consuming plant-based diets, but also in those consuming animal-based diets; hence, it is important to provide optimal education, food fortification, and adequate supplementation to achieve optimal nutritional/nutrient status among people with CKD.

In one systematic review [81], while dietary protein intake was lower in people consuming plant-based diets compared to those consuming animal-based diets, the overall dietary protein intake was well within the recommended intake levels for both groups, and dietary energy intake was comparable among those receiving plant-based vs. animal-based diets. Given that some nutrients are mainly present in and/or have greater bioavailability in plant-based or animal-based foods, some dietary patterns may lead to favorable intake of some nutrients yet inadequate intake of other nutrients. Plant-based diets typically have higher fiber, total PUFA, α-linolenic acid (ALA), vitamin B1, vitamin B6, vitamin C, vitamin E, folate, and magnesium contents, lower protein contents (albeit within recommended levels), and potentially lower eicosapentaenoic acid (EPA), docosahexaenoic acid (DHA), vitamin B12, vitamin D, calcium, iodine, iron (in women), and zinc contents. Taking vitamin B12 supplements or foods fortified with vitamin B12 is essential for people at risk of vitamin B12 deficiency, including those following vegan diets (owing to the absence of this vitamin in plant-based sources [89]) and people with CKD, who have reduced absorption of nutrients (age reduces absorption capacity), low intake of animal-based foods in a low-protein diet, and prescribed medications that can compromise the assimilation of vitamin B12 (e.g., proton-pump inhibitors and metformin) [90]. On the other hand, typical animal-based diets have higher protein, niacin, vitamin B12, and zinc contents, yet they may be inadequate with respect to fiber, total PUFA, ALA (in men), vitamin D, vitamin E, folate, calcium, and magnesium contents. Dietary monounsaturated fatty acids (MUFAs) can come from both plant-based and animal-based sources, but recent data have shown that MUFAs from plant-based foods have favorable associations with respect to lower risk of coronary heart disease [91] and mortality [92].

### 6.3. Protein Adequacy Overall and with Physical Activity

There has been a misconception that the nutritional quantity and quality of protein from plant-based diets are inferior to those of protein from animal-based foods. However, data from the general population do not support this impression. For example, landmark data from a cross-sectional analysis of 71,751 participants from the Adventist-Health-Study-2 showed that the median total protein intake did not differ among non-vegetarians (~75 g/day) vs. vegetarians (i.e., lacto-ovo vegetarians and vegans) (~71 g/day) [82]. A systematic review that included 141 observational and interventional studies, largely from Europe, South/East Asia, and North America, reported that the average dietary protein intake was lower in vegetarians and vegans compared to meat-eaters, but still within the recommended levels across these groups [81].

High-quality or complete protein sources for humans are dependent on whether the food contains adequate levels of indispensable amino acids to support human growth and/or is readily digested and absorbed [93]. According to the amino acid scoring system, which is currently the recommended method for evaluating dietary protein quality by the Food and Agricultural Organization of the United Nations (FAO) and the U.S. National Academy of Sciences, most animal proteins and soy proteins are generally considered to be complete protein sources [93]. Although individual plant proteins (except for soy protein) have insufficient levels of one or more indispensable amino acids, consumption of different sources of plant proteins over the course of the day can help to meet the requirements for indispensable amino acids, allowing them to be complete proteins and, hence, provide health benefits [83].

The topics of leucine content and muscle protein synthesis (MPS) have become popular in secular culture and among active individuals. Given the lower percentage of leucine in plant-based proteins (e.g., soy protein: ~8%) vs. animal proteins (e.g., whey protein: ~12%), there is a misconception that plant proteins are inferior to animal proteins with respect to attaining optimal lean body mass and muscle strength. Contrary to this hypothesis, a study examining differences in MPS at rest and following exercise followed by high-leucine/fast-digesting (hydrolyzed whey isolate), lower-leucine/intermediate-digesting (soy isolate), and high-leucine/slow-digesting (micellar casein) protein sources demonstrated that soy protein outperformed casein both at rest and post-exercise [84]. Neither soy nor caseins promoted greater post-exercise MPS than whey protein, and the post-exercise MPS fractional synthetic rate (%/h) for soy was still about 80% of that of whey. Moreover, MPS at rest after soy protein ingestion was similar to that after whey protein and higher than that after casein protein. Although some resistance training studies (duration 12–36 weeks) among young adults have reported better muscle mass and strength with fluid milk or whey protein [94,95], a meta-analysis of nine resistance training studies (duration 6 to 36 weeks) pooling together 266 participants, including both younger (18 to 38 years) and older (61 to 67 years) adults, showed no differences between soy protein and animal proteins with regards to improvements in bench press strength, squat/leg press strength, or lean body mass outcomes [85].

In terms of the effect of plant-based protein intake on risk of sarcopenia—the loss of skeletal muscle mass and physical function that occurs with advanced age—limited studies among non-CKD [96] and CKD populations [97] have reported that higher consumption of fruit and/or vegetables was correlated with a reduced risk of sarcopenia. Although these data and the comparable muscle-related benefits of plant-based protein compared to animal-based protein, as mentioned above, could mitigate concerns about developing sarcopenia following plant-based diets in people with advanced CKD, future studies evaluating the impact of plant-based diets vs. animal based-diets on muscle heath and sarcopenia, with consideration of overall diet quality and sufficient energy intake, are needed.

### 6.4. Soy Protein and Isoflavones

Given that soy protein contains isoflavones, which are compounds with a similar chemical structure to that of estrogen, it has been debated as to whether they provide health benefits or potential adverse effects (e.g., thyroid dysfunction, breast cancer). However, these concerns have largely stemmed from in vitro cell cultures or rodent studies involving large doses of isoflavones, and multiple lines of research over the past decade have not observed adverse hormonal effects from physiological amounts of soy foods in the diet [86].

### 6.5. Hyperkalemia

**Figure 3:**
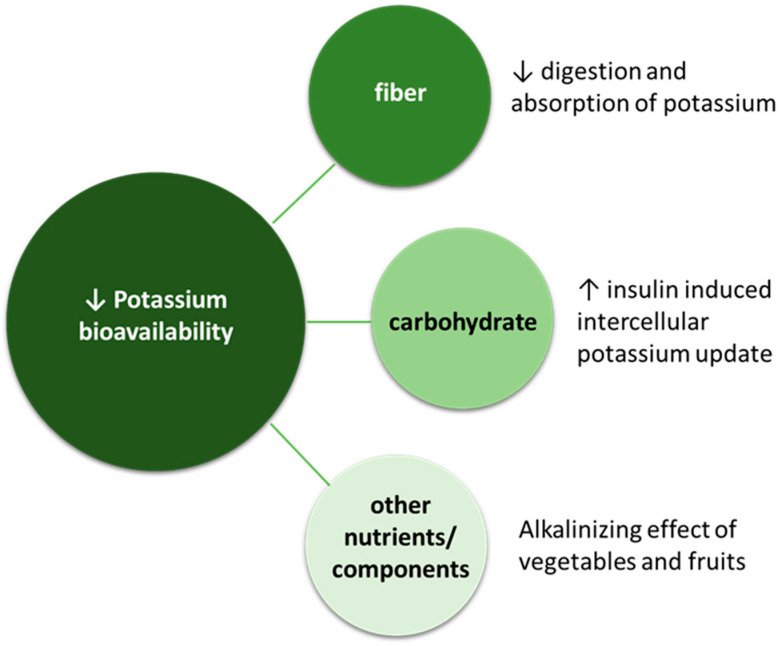
Potential mechanisms contributing to the lower bioavailability of potassium from plant-based foods. Abbreviations: K, potassium.

**Figure 4:**
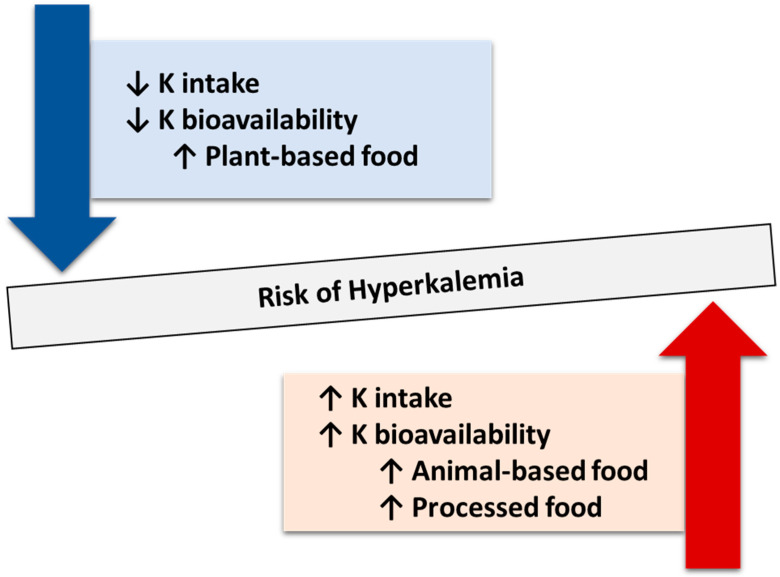
Potential dietary factors related to the risk of hyperkalemia. Lower absolute dietary potassium intake and/or having a diet with lower potassium bioavailability following consumption of healthy plant-based diets composed of unprocessed plant-based foods could result in a reduced risk of hyperkalemia. On the other hand, higher absolute dietary potassium intake and/or having a diet with higher potassium bioavailability following consumption of animal-based foods and processed foods could result in increased risk of hyperkalemia. Abbreviations: K, potassium.

There has been a longstanding paradigm in the clinical management of CKD/ESKD patients to avoid plant-based diets and/or fruits due to concerns regarding the risk of hyperkalemia. In a case review of 27 people with underlying CKD, acute kidney injury, or unspecified kidney disease, the majority of hyperkalemic episodes were related to the consumption of plant-based foods with higher bioavailability of potassium (e.g., juices, sauces, or dried fruits) vs. whole foods or unprocessed plant-based foods [88]. Similar to the bioavailability of dietary phosphorus, potassium from unprocessed plant-based foods has lower bioavailability than that of animal-based foods and processed foods. In a crossover feeding trial of 11 healthy men and women, the bioavailability of potassium from unprocessed fruits and vegetables was no more than 60% and lower than that of animal-based foods and fruit juices [98]. Another crossover feeding trial including six volunteers found that processed foods with potassium-containing additives resulted in 90 to 100% potassium bioavailability [99]. A similarly high bioavailability of 50–60% was found in a study of the DASH diet among 11 men and women with CKD over two weeks [32]. A differential association of dietary potassium intake from plant-based and animal-based diets with mortality risk was also found in the NHANES cohort. This study reported that, compared with high dietary potassium intake from plant-based foods, participants with low potassium intake from animal-based foods and pairings of low potassium intake with high protein, low fiber, or high phosphorus consumption were each associated with a higher mortality risk among 3172 participants with impaired kidney function [100]. One possible reason for the lower bioavailability of potassium in plant-based foods may be the increased intercellular potassium uptake induced by the insulin response to concomitant glucose, as well as slower and attenuated rises in serum potassium levels due to high dietary fiber content (Figure 3). Indeed, data from prospective observational and experimental studies, along with cross-sectional analyses examining varying proportions of plant contents, show that the occurrence of hyperkalemia is quite rare with plant-based diets (Figure 4) [87].

### 6.6. All-Cause Mortality

Growing data show that plant-based diets are associated with greater survival in the general population, as well as in CKD patients. In an analysis of 1065 people with eGFR < 60 mL/min/1.73 m^2^ from the NHANES study, each 33% increase in the proportion of plant protein to total protein intake was associated with a 23% lower mortality risk after a mean follow-up of 8.4 years [101]. Another analysis of the NHANES cohort also reported that higher total dietary protein intake of ≥1.4 g/kg actual body weight/day and the highest two tertiles of protein intake from animal-based foods were associated with a higher mortality risk among 1994 participants with impaired kidney function [102]. A study of 3972 people with CKD from the REGARDS study observed independent associations of southern and plant-based pattern scores with mortality risk after a mean 6.4 years of follow-up [71]. These results are in agreement with a meta-analysis of studies including 15,285 adults with CKD from seven cohorts, which showed that healthy dietary patterns (i.e., higher intake of fruit and vegetables, legumes, cereals, whole grains, and fiber) were associated with a lower risk of death [70].

## 7. Conclusions

In summary, incorporating vegetarian and plant-based diets using a personalized approach in the clinical management of CKD/ESKD not only provides health benefits to people with kidney disease, but also has the potential to maintain their nutritional status at optimal levels while avoiding the risk of PEW.

## Figures and Tables

**Table 1:** Different types of plant-based low-protein diets.

Diet	CKD Stage	Protein	Carbohydrates
LPD vegan	3–4	0.7 g/kg/day (100% from grain and legumes)	From cereals
LPDs vegan	3–4 Indicated in pregnant women with advanced CKD [9], in people at high risk of malnutrition, or in people who do not tolerate legumes [10]	0.6 g/kg/day (100% from cereals and legumes) + EAAs/KAs (1 tablet every 10 kg of body weight)	From cereals
PLADO diet	3–5	0.6 g/kg/day (with >50% plant-based sources)	From whole cereals
PLAFOND diet	3–5 Diabetic nephropathy	0.6 to <0.8 g/kg/day (with >50% plant-based sources)	From whole cereals
VLPDs	4–5	0.3–0.4 g/kg/day + EAAs/KAs (1 tablet every 5 kg of body weight)	Especially from low-protein substitutes

LPD: low-protein diet; LPDs: low-protein diet supplemented; PLADO: Plant-Dominant Low-Protein Diet; PLAFOND: patient-centered plant-focused LPD for the nutritional management of CKD/DM; VLPDs: very-low-protein diet supplemented. EAAs/KAs: essential amino acids/keto acids.

**Table 2:** Importance of dietary fiber in human health.

Property	Function	Health Benefits
**Bulk**	Adds bulk to diet ○ Satiety effect Adds bulk to stool ○ Improves GI motility ▪ Increases bowel movement ▪ Reduces intestinal transit time ○ Increases fecal bulk ○ Increases stool frequency	Regulates energy intake Lowers blood pressure Promotes weight loss Alleviates constipation
**Viscosity**	Inhibits intestinal digestion and absorption ○ Inhibits glucose absorption ▪ Traps carbohydrates ▪ Slows glucose absorption ○ Inhibits cholesterol absorption ▪ Traps bile acids and extracts to feces ▪ Increases the synthesis of bile acids from cholesterol ○ Traps carcinogenic substances	Improves glycemic control ○ Lowers postprandial serum glucose levels Improves cholesterol control ○ Lowers serum total and LDL cholesterol Contributes to cancer prevention
**Fermentability**	Alters intestinal microbiota composition and function ○ Increases gut-microbiome-induced production of SCFAs	Anti-inflammation Anti-obesity Anti-diabetes Anticancer Hepatoprotection Cardiovascular protection Neuroprotection Constipation treatment Inflammatory bowel disease treatment Immunoregulation

Abbreviations: GI, gastrointestinal; LDL, low-density lipoprotein; SCFAs, short-chain fatty acids.

**Table 3:** Common concerns/myths and existing evidence with respect to plant-based diets.

Topic	Concern/Myth	Evidence
**Nutritional adequacy**	Plant-based diets lack adequate contents of nutrients largely found in animal-based foods	Dietary energy intake is similar across dietary patterns, and protein intake seems to be lower in people following plant-based diets compared to those following animal-based diets, but still well within the recommended intake levels [81] There are nutrient inadequacies across all dietary patterns, including plant-based diets and animal-based diets
**Protein adequacy**	Plant-based diets provide inferior protein quantity compared to animal-based diets	Plant-based diets are not low-protein diets per se Large-population-based data have not shown differences in dietary protein intake across plant-based vs. animal-based diets [82]
	Plant-based diets provide inferior protein quality compared to animal-based diets	Although individual plant proteins (except for soy protein) have insufficient levels of one or more indispensable amino acids, consumption of different sources of plant proteins over the course of the day can help to meet the requirements for indispensable amino acids and allow them to be complete proteins and provide health benefits [83]
	Plant proteins are inferior to animal proteins in terms of lean body mass and strength	There is a lower percentage of leucine in plant proteins (e.g., soy protein: ~8%) than in animal proteins (e.g., whey protein: ~12%) Muscle protein synthesis (MPS) [84] ○ Soy protein promotes greater MPS at rest and post-exercise (vs. casein protein) ○ Soy protein promotes comparable MPS at rest and 20% lower MPS post-exercise (vs. whey protein) No differences between soy protein and animal proteins for improvements in bench press strength, squat/leg press strength, or lean body mass [85]
**Hormonal abnormalities**	Isoflavones from soy have potential adverse effects (e.g., thyroid dysfunction, breast cancer)	Concerns have been raised largely based on in vitro cell cultures or rodent studies involving large doses of isoflavones Studies have not observed adverse hormonal effects from physiological amounts of soy foods in the diet [86]
**Hyperkalemia**	Plant-based diets cause hyperkalemia	The occurrence of hyperkalemia is quite rare [87] The majority of hyperkalemic episodes seem to be related to the consumption of plant-based foods containing higher bioavailable potassium contents (e.g., juices, sauces, or dried fruits) compared with whole foods or unprocessed plant-based foods [88]

Abbreviations: MPS, muscle protein synthesis.

## Data Availability

Not applicable.
